# The relationship between appetite and food preferences in British and Australian children

**DOI:** 10.1186/s12966-015-0275-4

**Published:** 2015-09-17

**Authors:** Alison Fildes, Kimberley M. Mallan, Lucy Cooke, Cornelia HM van Jaarsveld, Clare H. Llewellyn, Abigail Fisher, Lynne Daniels

**Affiliations:** Health Behaviour Research Centre, Department of Epidemiology and Public Health, University College London, Gower Street, London, WC1E 6BT UK; Institute of Health and Biomedical Innovation, School of Exercise and Nutrition Sciences, Queensland University of Technology, Brisbane, Queensland Australia; Department of Primary Care and Public Health Sciences, King’s College London, Capital House, 42 Weston Street, London, SE1 3QD UK; School of Psychology, Australian Catholic University, Brisbane, Queensland Australia

**Keywords:** Appetite, Food preference, Eating behaviour, Obesity, Children, Diet

## Abstract

**Background:**

Appetitive traits and food preferences are key determinants of children’s eating patterns but it is unclear how these behaviours relate to one another. This study explores relationships between appetitive traits and preferences for fruits and vegetables, and energy dense, nutrient poor (noncore) foods in two distinct samples of Australian and British preschool children.

**Methods:**

This study reports secondary analyses of data from families participating in the British GEMINI cohort study (*n* = 1044) and the control arm of the Australian NOURISH RCT (*n* = 167). Food preferences were assessed by parent-completed questionnaire when children were aged 3–4 years and grouped into three categories; vegetables, fruits and noncore foods. Appetitive traits; e*njoyment of food, food responsiveness*, *satiety responsiveness*, *slowness in eating*, and *food fussiness* were measured using the Children’s Eating Behaviour Questionnaire when children were 16 months (GEMINI) or 3–4 years (NOURISH). Relationships between appetitive traits and food preferences were explored using adjusted linear regression analyses that controlled for demographic and anthropometric covariates.

**Results:**

Vegetable liking was positively associated with *enjoyment of food* (GEMINI; β = 0.20 ± 0.03, *p* < 0.001, NOURISH; β = 0.43 ± 0.07, *p* < 0.001) and negatively related to *satiety responsiveness* (GEMINI; β = -0.19 ± 0.03, *p* < 0.001, NOURISH; β = -0.34 ± 0.08, *p* < 0.001), *slowness in eating* (GEMINI; β = -0.10 ± 0.03, *p =* 0.002, NOURISH; β = -0.30 ± 0.08, *p* < 0.001) and *food fussiness* (GEMINI; β = −0.30 ± 0.03, *p* < 0.001, NOURISH; β = -0.60 ± 0.06, *p* < 0.001). Fruit liking was positively associated with *enjoyment of food* (GEMINI; β = 0.18 ± 0.03, *p* < 0.001, NOURISH; β = 0.36 ± 0.08, *p* < 0.001), and negatively associated with *satiety responsiveness* (GEMINI; β = −0.13 ± 0.03, *p* < 0.001, NOURISH; β = −0.24 ± 0.08, *p* = 0.003), *food fussiness* (GEMINI; β = -0.26 ± 0.03, *p* < 0.001, NOURISH; β = −0.51 ± 0.07, *p* < 0.001) and *slowness in eating* (GEMINI only; β = -0.09 ± 0.03, *p =* 0.005). *Food responsiveness* was unrelated to liking for fruits or vegetables in either sample but was positively associated with noncore food preference (GEMINI; β = 0.10 ± 0.03, *p* = 0.001, NOURISH; β = 0.21 ± 0.08, *p* = 0.010).

**Conclusion:**

Appetitive traits linked with lower obesity risk were related to lower liking for fruits and vegetables, while *food responsiveness*, a trait linked with greater risk of overweight, was uniquely associated with higher liking for noncore foods.

**Electronic supplementary material:**

The online version of this article (doi:10.1186/s12966-015-0275-4) contains supplementary material, which is available to authorized users.

## Introduction

A quarter or more of children in developed countries such as Australia [1] and the UK [2] are classified as overweight or obese. The aetiology of obesity is complex, with both genetic and environmental factors contributing to excess weight gain in childhood. Dietary behaviours thought to contribute to childhood obesity include appetitive traits (such as responsiveness to food cues and sensitivity to feelings of fullness) which influence the ‘quantity’ of children’s food intake [3], and food preferences (the extent to which an individual likes particular foods) which contribute to dietary ‘quality’ [4]. However the relationships between these independent risk factors have rarely been explored. Given that overweight and obesity in childhood track into adulthood [5] and carry significant negative social, emotional and physiological consequences [6–9], there is a clear need to improve our understanding of early life obesogenic behaviours.

Appetitive traits and food preferences have previously been identified as separate and important predictors of children’s eating [3, 10]. Children’s food preferences generally do not align with dietary recommendations [11], and when identifying their most and least favourite foods, children typically rate fatty and sugary foods as the most liked and vegetables the least liked [12]. Children with stronger appetitive traits, such as higher food responsiveness (eating in response to food cues, e.g. the smell or sight of food) or lower satiety sensitivity (decreased sense of satisfaction and fullness after eating), are more likely to overeat in response to palatable food [13]. Longitudinal studies suggest that appetitive traits and food preferences established in infancy and early childhood are likely to persist into adulthood [14–16]. Insight into the relationships between these behavioural influences on young children’s dietary patterns could inform strategies for improving healthy nutrition and growth**.**

To date, research on children’s food preferences has largely focused on aspects of the family food environment, such as exposure or modelling [17–20], but characteristics of the child are also likely to influence individual patterns of food likes and dislikes. Food neophobia, the predisposition for rejecting novel or unknown foods, is a normal developmental phase for young children that typically peaks between 2 and 6 years of age [21]. Children who are more neophobic tend to show lower preference for and intake of vegetables most commonly [22–24]. The related construct of food fussiness (or pickiness) has also been linked with lower dietary variety and quality. Fussier children, in addition to refusing new foods may also resist eating many familiar – but usually less popular – foods and typically have a very narrow range of foods that they are prepared to eat [25]. Like neophobia, fussiness has been linked with decreased consumption of and preferences for plant-based foods, particularly vegetables [25–27].

Children’s appetitive traits as measured by the Children’s Eating Behaviour Questionnaire (CEBQ) [28] have been most often examined in terms of their relationship to (excess) energy intake and weight [13, 29–31], but there is emerging evidence to indicate that some of these traits may be linked with patterns of food preferences [32–34]. These appetitive traits have been broadly categorised as food ‘approach’ or ‘avoidance’. The ‘food approach’ trait *enjoyment of food* (capturing the amount of pleasure experienced when eating) has been linked with higher fruit and vegetable intake [32] but has not been studied in relation to other food groups. As *enjoyment of food* has been shown to associate positively with weight in childhood [30], insight into its relationship with liking for noncore foods (energy dense, nutrient poor discretionary foods such as chocolate or chips) would facilitate further understanding of this trait. Similarly, a second ‘food approach’ trait, *food responsiveness* (responsiveness to external food cues) has also been linked to higher weight status in childhood [13, 30], while ‘food avoidance’ appetitive traits *satiety responsiveness* (sensitivity to feelings of fullness) and *slowness in eating* (slower eating speed) have been negatively related to weight [30].

When considering the mechanism (s) that underlie the association between appetitive traits and weight status, researchers have tended to focus on ‘how much’ rather than ‘what’ children eat. However, whether appetitive traits relate to the quality, not just quantity, of children’s diets through associations with patterns of preferences for ‘healthy’ and ‘unhealthy’ foods has yet to be systematically explored. The present study aims to investigate the relationships between multiple appetitive traits and preferences for fruits and vegetables and noncore foods in two distinct samples of young children from two different countries (Australia and Britain) and hence different food environments. It was of interest whether particular food approach (*enjoyment of food*, *food responsiveness*) and avoidance (*satiety responsiveness*, *slowness in eating*, *food fussiness*) behaviours would be associated with children’s preference for fruits, vegetables and noncore foods. Based on previous findings, *food fussiness* is predicted to associate with decreased preferences for fruits and vegetables [25]. While the food approach traits, *enjoyment of food* and *food responsiveness,* are expected to be associated with higher preference for noncore foods.

### Methods

#### Study design

This study reports secondary analysis of data from two studies: the UK GEMINI twin study [35] and the Australian NOURISH randomized controlled trial (RCT) [36]. The purpose of including both samples was to enhance the generalizability of the findings by looking for similar patterns of associations (i) in twins (GEMINI) and singletons (NOURISH), (ii) using longitudinal (GEMINI) and cross-sectional data (NOURISH), and (iii) across two different food and feeding environments.

#### Participants

### Sample 1: GEMINI

Sample 1 was drawn from the GEMINI twin study. GEMINI is a population-based cohort of UK twins born in 2007 [35]. Participants were recruited by the Office for National Statistics who contacted all families with twins born in England and Wales between March and December 2007 (*N* = 6754); of whom 2402 (36 %) completed the baseline questionnaire and consented to participate. Baseline questionnaires were completed when children were approximately 8 months old. CEBQ data were collected when children were 16 ± 1 months, and food preference data when they were 42 ± 3 months (3.5 years). Ethical approval for GEMINI was granted by the Joint University College London/University College London Hospitals Committee on the Ethics of Human Research. One twin from each family, for whom complete data were available on all the study variables, was selected at random for inclusion in the analyses (*n* = 1044).

### Sample 2: NOURISH

Sample 2 comprised Australian children who were allocated to the control condition of the NOURISH RCT [36]. NOURISH is an early feeding intervention that enrolled 698 first-time mothers in two Australian cities (Brisbane and Adelaide) between February 2008 and March 2009. All participating mothers were healthy, primiparous English-speaking women with infants who were healthy at birth (>35 weeks, >2500 g). Baseline questionnaires were completed when children were approximately 4 months old. CEBQ and food preference data were collected when children were 44 ± 3 months (3.7 years). Approval for the NOURISH study was obtained from 11 Human Research Ethics Committees covering Queensland University of Technology, Flinders University and all the recruitment hospitals (QUT HREC 00171 Protocol 0700000752). The trial was registered with the Australian and New Zealand Clinical Trials Registry Number (ACTRN) 12608000056392. All control group children with complete data on the study variables were included in the present analyses (*n* = 167).

#### Measures

### Food preference scales

The food preference scales used in GEMINI and NOURISH were collected using an established tool [12, 37]. The development of the food preference scales used in the GEMINI study has been described previously [38]. Briefly, parents reported their child’s preference for a large number of individual foods using a 6 point scale with response options of: ‘likes a lot’, ‘likes’, ‘neither likes or dislikes’, ‘dislikes’, ‘dislikes a lot’, and ‘never tried’ (the last recoded to missing). Responses were scored 1–5 with higher scores indicating higher liking. Foods tried by at least 75 % of the children were grouped into categories primarily based on a principal components analysis [38]. Three of these food categories, vegetables (eg, broccoli, green beans, sweet potato, and parsnips), fruits (eg, banana, strawberries, pear and mango) and noncore foods (eg, chocolate, cookies, ice cream, and chips i.e. high fat and/or sugar and energy density) are the focus of the present study. Cronbach’s α for the food-group scales showed an acceptable internal reliability for vegetables (α = 0.88; 19 items), fruit (α = 0.88; 16 items) and noncore foods (α = 0.76; 12 items).

The food preference scales used in GEMINI were adapted for the NOURISH sample by changing some of the individual food items to better reflect commonly consumed Australian (rather than British) foods. Parents similarly reported their child’s liking for fruits, vegetables and noncore food items using the same 6-point response scale. Individual foods that had been tried by 75 % of children were grouped into comparable vegetables (eg, broccoli, green beans, pumpkin, and zucchini), fruits (eg, banana, peaches, pineapple and kiwi) and noncore snacks (eg, cake, potato crisps, ice cream, and chips) categories. Cronbach’s α for the NOURISH food-group scales again showed acceptable internal reliability; vegetables (α = 0.90; 18 items) and fruits (α = 0.86; 12 items) and was somewhat lower for noncore foods (α = 0.64; 9 items).

In GEMINI and NOURISH samples preference data were collected at 42 ± 3 months (3.5 years) and 44 ± 3 months (3.7 years) respectively. Scale scores were calculated as the mean liking for component food items. Participants were required to have completed more than half of items within each scale for a score to be calculated.

### Appetitive traits

The CEBQ is a 35 item tool designed to assess traits that have been implicated in the development of overweight [28]. The CEBQ has eight scales in total, but only five were used in the present analyses: *enjoyment of food* (4 items, e.g., *My child enjoys eating*); *food responsiveness* (5 items, e.g., *My child’s always asking for food*); *satiety responsiveness* (5 items, e.g., *My child gets full up easily*); *slowness in eating* (4 items, e.g., *My child eats slowly*); and *food fussiness* (6 items, e.g., *My child refuses new foods at first).* The individual CEBQ scale items are listed in an additional table. Items were scored on a 5-point scale as ‘never’, ‘rarely’, ‘sometimes’, ‘often’, or ‘always’. Mean scores were calculated for each subscale (range: 1–5) with higher scores indicating higher values of each trait. In order to calculate subscale scores complete data was required on a minimum of 60 % of scale items.

NOURISH parents completed the CEBQ [28]at the same time as the food preference measures (i.e. 44 ± 3 months) whereas GEMINI parents completed the CEBQ when their children were 16 ± 1 months, approximately 2 years before they completed the food preference measures (42 ± 3 months). In the GEMINI sample a minor modification was made to the CEBQ in order for it to be age-appropriate for toddlers: one item from the *food responsiveness* scale (‘If given the chance, my child would always have food in his/her mouth’) was omitted. All other CEBQ scales remained unchanged (see Additional file 1: Table S1).

### Demographic characteristics

Data on child gender, age (months), gestational age, and maternal age and education were collected for each sample at the time of the baseline questionnaires. Maternal education was dichotomised as below university education versus a minimum of undergraduate university education. All NOURISH children were firstborns. The parity of GEMINI mothers was assessed using the question: ‘How many other children live in the home with your twins’ and was categorised as none, or one or more older children. In both samples mothers reported separately on the frequency of fruit and vegetable servings that they themselves consumed. In the GEMINI sample these questions referred to the number of servings each of fruits and vegetables consumed in the past week. In the NOURISH sample mothers reported their consumption of fruits and vegetables by responding to the question ‘how many serves of fruit [or vegetables] do you usually eat each day’. For both samples the data were recoded to provide an estimation of the total number each of fruit and vegetable portions consumed daily.

Questions on children’s age at which solid foods were introduced were asked on two occasions. In GEMINI, parents were asked, ‘at what age did your twins start taking solid foods every day’ (separate response for each child). This was asked as part of the baseline questionnaire (at around 8 months) and again when the children were 16 months. Where possible, responses were taken from baseline to ensure responses were given closer to the time of food introduction. In the NOURISH sample, age at first solids was assessed at baseline (at around 4 months) and again at 14 months with the question ‘at what age was your child first given solid or semi-solid food regularly’. As the majority of NOURISH children had not yet started solids at baseline, responses were taken from the 14 month questionnaire.

In GEMINI, feeding method was assessed with the question: ‘Which feeding methods did you use in the first three months’, with responses categorised as: (1) ‘breastfed’ (‘entirely breastfeeding’ or ‘mostly breastfeeding with some bottle-feeding’) and (2) ‘mixed or formula-fed’ (all other categories). In NOURISH, mothers were asked at baseline; ‘How are you currently feeding your baby’, which was categorised into: 1) ‘breastfed’ (‘exclusive breastfeeding’ or ‘breastfeeding fully with occasional water and juices’), and 2)’mixed or formula-fed’ (‘combination breast and formula feeding’ or ‘formula feeding only’).

Child weight and height for NOURISH participants were measured by trained research staff [36]. In GEMINI, parents copied health professional weights and heights that were recorded in the ‘red book’ in the early months. They were also sent weighing scales and growth charts to the home. For both samples, exact age at weight measurement was calculated. All measurements were converted to a BMI-for-age *Z*-score (BMIZ) using the World Health Organization [39] Anthro software program version 3.0.1 and macros.

### Statistical analyses

All analyses were conducted separately for the two samples. Separate unadjusted linear regression analyses were conducted first to identify significant univariate relationships between each appetitive trait and preferences for each of the three food groups (vegetables, fruit and noncore foods). The CEBQ scales were then entered into adjusted linear regression models as independent variables to investigate whether associations remained unchanged when controlling for demographic and anthropometric factors (listed in Table 1). Child sex, weight, age at introduction of solid foods, method of milk feeding, and maternal education and maternal fruit and vegetable intake have all previously been associated with children’s food preferences and/or appetitive traits and were therefore included as covariates in the adjusted models [12, 28, 32, 40–42]. There were no substantive differences between results of the unadjusted and adjusted analyses, therefore only the adjusted results are presented. All analyses were performed in SPSS Version 21 for Windows. A significance level of *p* < 0.05 was applied throughout.

**Table 1:** Sample Characteristics

	GEMINI (*n* = 1044) [Britain]	NOURISH (*n* = 167) [Australia]
	Mean (SD) or N (%)	Mean (SD) or N (%)
*Demographics*		
Gender (male)	506 (48 %)	76 (46 %)
Maternal education (tertiary undergraduate and above)	535 (51 %)	115 (69 %)
Child BMI Z-score^a^	0.4 (1.1)	0.6 (0.9)
Age at weight assessment (years)	3.5 (0.22)	3.7 (0.3)
Gestational age (in weeks)	36.3 (2.47)	all >35 weeks
Parity (primapara)	592 (57 %)	167 (100 %)
Feeding method (breast)	402 (39 %)	98 (59 %)
Age at solid food introduction (months)	5.0 (1.0)	5.3 (1.1)
Maternal fruit intake (portions per day)	1.7 (1.1)	1.7 (0.9)
Maternal vegetable intake (portions per day)	2.2 (1.1)	2.6 (1.1)
*Food preference scales* ^b^		
Vegetables	3.4 (0.6)	3.4 (0.9)
Fruits	4.0 (0.7)	4.2 (0.7)
Noncore foods	4.4 (0.4)	4.7 (0.4)

^a^BMI-for-age Z-scores (BMIZ) were calculated from height and weight data and exact age at measurement using the World Health Organization [36] Anthro software program version 3.0.1 and macros

^b^Mean liking scores for food group scales (comprised of multiple single food preference items) [35] with higher scores indicating higher liking

## Results

Characteristics of the two samples are shown in Table 1. Just under half of the children in GEMINI (48 %) and NOURISH (46 %) were male. The proportion of mothers with a university education was 51 % in GEMINI and 69 % in NOURISH. In GEMINI 39 % of children were ever breastfed compared to 59 % of NOURISH children. Age at introduction of solid foods was around 5 months in both GEMINI (5.0 months) and NOURISH (5.3 months). GEMINI children had a mean gestational age of 36 weeks, while all NOURISH children were born at greater than 35 weeks gestation. In both samples of children, liking was lowest for vegetables (GEMINI; 3.4 ± 0.6, NOURISH; 3.4 ± 0.9) and highest for noncore foods (GEMINI; 4.4 ± 0.4, NOURISH; 4.7 ± 0.4), with fruit also well liked (GEMINI; 4.0 ± 0.7, NOURISH; 4.2 ± 0.7). The means (and standard deviations), Cronbach’s alphas and correlations for the CEBQ scale scores for both samples are shown in Table 2. Each of the CEBQ scales were significantly correlated with one another, with the exception of *food fussiness* and *food responsiveness* in the NOURISH sample. These two scales also showed the weakest (negative) correlation in the GEMINI sample. The two ‘food approach’ scales *enjoyment of food* and *food responsiveness* correlated positively with one another and negatively with the ‘food avoidance’ scales *satiety responsiveness* and *slowness in eating* in both samples.

**Table 2:** Child eating behaviour scales [28] in GEMINI and NOURISH

	Mean (SD)	Cronbach’s Alpha (α)	Correlations				
			Enjoyment of food	Food responsiveness	Satiety responsiveness	Slowness in eating	Food fussiness
GEMINI (*n* = 1044)^a^							
Enjoyment of food	4.17 (0.60)	0.85	1	.344**	-.636**	-.458**	-.620**
Food responsiveness	2.22 (0.75)	0.76		1	-.444**	-.260**	-.168**
Satiety responsiveness	2.70 (0.63)	0.78	-	-	1	.574**	.470**
Slowness in eating	2.48 (0.64)	0.67	-	-	-	1	.340**
Food fussiness	2.19 (0.69)	0.86	-	-	-	-	1
NOURISH (*n* = 167)^b^							
Enjoyment of food	3.77 (0.67)	0.89	1	.378**	-.510**	-.505**	-.664**
Food responsiveness	2.42 (0.68)	0.77	-	1	-.311**	-.242**	-.105
Satiety responsiveness	2.99 (0.58)	0.78	-	-	1	.503**	.374**
Slowness in eating	3.09 (0.72)	0.82	-	-	-	1	.463**
Food fussiness	2.87 (0.82)	0.93	-	-	-	-	1

**Correlation is significant at the 0.01 level (2-tailed)

^a^The CEBQ scale data were collected in GEMINI when children were 16 ± 1 months

^b^The CEBQ scale data were collected in NOURISH when children were 44 ± 3 months (3.7 years)

### Vegetable preference

Associations between each of the CEBQ scales and vegetable preference score, controlling for demographic and anthropometric variables, are shown in Table 3 for GEMINI and NOURISH. In both samples vegetable liking was positively associated with *enjoyment of food*, and negatively related to *satiety responsiveness*, *slowness in eating* and *food fussiness. Food fussiness* showed a particularly strong negative relationship with vegetable liking, and explained the largest amount of variance among both GEMINI (9 %; sr^2^ = .089, *p* < .001) and NOURISH (34 %; sr^2^ = .340, *p* < .001) children. *Food responsiveness* was not related to liking for vegetables in either the GEMINI or NOURISH samples.

**Table 3:** Factors associated with vegetable preference^a^

	Unstandardized Beta (SE)	Standardized Beta (SE)	*p* value	R^2^	sr^2^
GEMINI (*n* = 1044)^b^					
Enjoyment of Food	**.204 (.031)**	**.202 (.031)**	**<.001**	**.071**	**.039**
Food Responsiveness	.027 (.026)	.033 (.031)	.294	.033	.000
Satiety Responsiveness	**-.182 (.030)**	**-.189 (.031)**	**<.001**	**.066**	**.033**
Slowness in Eating	**-.092 (.029)**	**-.097 (.031)**	**.002**	**.041**	**.009**
Food Fussiness	**-.267 (.026)**	**-.302 (.029)**	**<.001**	**.122**	**.089**
NOURISH (*n* = 167)					
Enjoyment of Food	**.544 (.092)**	**.426 (.072)**	**<.001**	**.240**	**.168**
Food Responsiveness	.142 (.102)	.113 (.082)	.167	.086	.004
Satiety Responsiveness	**-.486 (.110)**	**-.335 (.076)**	**<.001**	**.177**	**.101**
Slowness in Eating	**-.357 (.088)**	**-.304 (.075)**	**<.001**	**.163**	**.088**
Food Fussiness	**-.632 (.066)**	**-.604 (.063)**	**<.001**	**.415**	**.340**

^a^Models adjusted for covariates as defined in Table 1 including sex, milk feeding method, age at first solids, maternal education, maternal fruit intake, maternal vegetable intake, BMI Z-score [36] and age at anthropometric measurements

^b^Models also adjusted for parity and gestational age (GEMINI sample only) Significant values (at an alpha level of *p* < 0.05) are **bolded**

### Fruit preference

Associations between appetitive traits and liking for fruit are shown in Table 4 and displayed a similar pattern to vegetables. In both samples, *enjoyment of food* was positively associated with fruit liking, while *satiety responsiveness* and *food fussiness* were negatively associated with liking. The negative association between *food fussiness* and fruit preference explained a sizeable proportion of the variance in this trait (GEMINI: 7 %; sr^2^ = .065, *p* < .001 and NOURISH: 24 %; sr^2^ = .239, *p* < .001). A significant negative relationship was observed between *slowness in eating* and fruit liking in the GEMINI sample only. No significant association was observed between *food responsiveness* and liking for fruits in either study sample.

**Table 4:** Factors associated with fruit preference^a^

	Unstandardized Beta (SE)	Standardized Beta (SE)	*p* value	R^2^	sr^2^
GEMINI (*n* = 1044)^b^					
Enjoyment of Food	**.193 (.033)**	**.179 (.031)**	**<.001**	**.078**	**.031**
Food Responsiveness	.052 (.027)	.059 (.031)	.056	.039	.003
Satiety Responsiveness	**-.135 (.032)**	**-.132 (.031)**	**<.001**	**.063**	**.016**
Slowness in Eating	**-.089 (.031)**	**-.088 (.031)**	**.005**	**.055**	**.007**
Food Fussiness	**-.242 (.028)**	**-.257 (.030)**	**<.001**	**.112**	**.065**
NOURISH (*n* = 167)					
Enjoyment of Food	**.374 (.077)**	**.363 (.075)**	**<.001**	**.185**	**.122**
Food Responsiveness	.147 (.083)	.145 (.082)	.078	.082	.018
Satiety Responsiveness	**-.281 (.092)**	**-.240 (.079)**	**.003**	**.115**	**.052**
Slowness in Eating	-.109 (.074)	-.115 (.079)	.102	.076	.012
Food Fussiness	**-.428 (.058)**	**-.507 (.069)**	**<.001**	**.303**	**.239**

^a^Models adjusted for covariates as defined in Table 1 including sex, milk feeding method, age at first solids, maternal education, maternal fruit intake, maternal vegetable intake, BMI Z-score [36] and age at anthropometric measurements

^b^Models also adjusted for parity and gestational age (GEMINI sample only)

Significant values (at an alpha level of *p* < 0.05) are **bolded**

### Non-core food preference

Associations between noncore food preference and CEBQ scales are shown in Table 5. Liking for noncore foods was positively associated with *food responsiveness* in both GEMINI and NOURISH samples. In GEMINI there was also a small but significant association between noncore foods and *enjoyment of food.*

**Table 5:** Factors associated with noncore food preference^a^

	Unstandardized Beta (SE)	Standardized Beta (SE)	*p* value	R^2^	sr^2^
GEMINI (*n* = 1044)^b^					
Enjoyment of Food	**.049 (.022)**	**.071 (.031)**	**.023**	**.032**	**.005**
Food Responsiveness	**.056 (.017)**	**.101 (.031)**	**.001**	**.037**	**.010**
Satiety Responsiveness	-.026 (.021)	-.039 (.032)	.213	.029	.001
Slowness in Eating	-.017 (.020)	-.026 (.031)	.400	.028	.001
Food Fussiness	-.022 (.019)	-.036 (.031)	.251	.029	.001
NOURISH (*n* = 167)					
Enjoyment of Food	.075 (.044)	.137 (.080)	.089	.062	.017
Food Responsiveness	**.114 (.044)**	**.212 (.082)**	**.010**	**.084**	**.041**
Satiety Responsiveness	.032 (.051)	.052 (.082)	.528	.047	.002
Slowness in Eating	-.021 (.040)	-.042 (.080)	.603	.046	.002
Food Fussiness	-.065 (.036)	-.145 (.080)	.072	.064	.020

^a^Models adjusted for covariates as defined in Table 1 including sex, milk feeding method, age at first solids, maternal education, maternal fruit intake, maternal vegetable intake, BMI Z-score [36] and age at anthropometric measurements

^b^Models also adjusted for parity and gestational age (GEMINI sample only)

Significant values (at an alpha level of *p* < 0.05) are **bolded**

The Beta scores (standardized regression coefficients) were consistently higher for all significant food preference and appetitive trait associations in the NOURISH sample compared with the GEMINI sample.

## Discussion

Each of the appetitive traits measured at either 16 months (GEMINI) or 3–4 years (NOURISH) were related to children’s food preferences measured at around four years of age. Associations varied across traits and food groups. As predicted, *food fussiness* was strongly inversely associated with both vegetable and fruit preference, but showed no association with noncore food preference. The two ‘food approach’ appetitive traits that have previously been linked with higher risk of overweight [13], were both positively associated with liking: *enjoyment of food* was related to greater liking for fruits and vegetables, while *food responsiveness* was related only to liking for noncore foods. In a counterintuitive finding, the two ‘food avoidance’ appetitive traits, *satiety responsiveness* and *slowness in eating,* that have previously been associated with lower risk of overweight [30] were related to lower liking for vegetables and fruits but showed no associations with noncore food liking. Taken together these findings suggest that the reported associations between higher ‘food avoidance’ appetitive traits and lower weight are not simply reflecting a pattern of lower caloric intake driven by decreased preference for noncore foods. Similarly, the relationship between ‘food approach’ traits and higher weight cannot easily be explained by poorer diet quality determined by increased preferences for noncore foods and/or lower preference for nutrient-dense foods such as fruits and vegetables.

Our findings provide some evidence that appetitive traits associated with increased risk of childhood overweight may have additional consequences for dietary diversity and nutrient intake. While *enjoyment of food* was found to relate to greater liking for fruits and vegetables, the other ‘food-approach’ trait *food responsiveness* (the drive to eat in response to the sight or smell of palatable food) was uniquely related to liking for noncore foods. The strong relationship between *enjoyment of food* and liking for both vegetables and fruits, is consistent with previous research linking greater *enjoyment of food* with higher intakes of fruits and vegetables [32]. In contrast, *food responsiveness* was singularly associated with noncore preference among both GEMINI and NOURISH children. This suggests children who were rated highly on *food responsiveness* are not only potentially at risk of higher adiposity, as has been reported previously [30, 43, 44], but may be specifically at risk of an unhealthy diet characterised by high noncore food intake.

The negative relationships observed between s*atiety responsiveness* and *slowness in eating,* and liking for vegetables and fruits, suggests that children who are less avid eaters display lower liking for nutrient-dense foods in particular. Interestingly these food avoidance traits did not manifest in lower preferences for noncore foods. Children displaying appetitive traits that place them at lower risk of overweight may not simply be consuming less overall, they may also be eating disproportionately fewer fruits and vegetables by virtue of their preferences. Future work that measures the dietary intake patterns of these food avoidant children is needed to test this proposition.

There was no significant association between *food fussiness* and liking for noncore foods in either NOURISH or GEMINI children. The weaker relationship between *food fussiness* and liking for noncore foods compared with core foods such as fruits and vegetables is consistent with other evidence. A previous study from NOURISH found that neophobia was related to lower liking for fruit and vegetables, but not noncore foods, at age two years [42]. The same pattern of associations was reported for intake among two to six year olds [22, 23]. In contrast, a recent study using NOURISH data (also at age two years) showed a positive association between food neophobia and proportion of daily energy intake from noncore foods [45]. An earlier study similarly reported higher consumption of sweetened foods among fussier children [46]. Findings that fussy children consume relatively more sweet or energy dense foods compared to more nutrient dense foods such as fruits and vegetables is consistent with the preference patterns for these foods. Also potentially relevant are carers’ responses to *food fussiness* that may include using well-liked noncore foods as a reward for eating less preferred foods or the ‘as long as they eat something’ approach whereby favourite foods are offered as alternatives to rejected foods. Overall, these food preference patterns and fussy behaviours potentially lead to over-consumption of highly palatable energy-dense foods through rejection of nutrient-dense foods and reduced dietary variety. This could put fussy children at risk of excessive future weight gain, although existing prospective studies have thus far failed to support this [28, 47]. The literature would benefit from further investigations with large prospective cohorts, using reliable and objective measures of fussy eating and direct impact on actual consumption to investigate long-term associations with weight status.

This is one of the first studies to explore the relationship between appetitive traits and food preferences. However due to the cross-sectional design, and without measures of food intake, we can only speculate on the causal direction of the associations observed. One potential causal mechanism driving the association between appetitive traits and food preferences is taste exposure. A large body of research has clearly demonstrated that repeated exposure to the taste of a food results in increased liking [17]. Fussier children are more likely to reject foods (such as vegetables), leading to fewer taste exposures and potentially lower preferences for these foods later on. Similarly a child who scores high on measures of ‘food approach’ behaviours may seek out and try new foods, ultimately leading to increased liking. Foods that are intrinsically liked from the start, such as sugary, energy dense noncore foods, would arguably be less susceptible to the effects of appetite driven exposure. A second explanation for the observed relationships between appetitive traits and food preferences is parental perception. A child who likes core foods such as fruits and vegetables may be perceived by parents as easier to feed and thus reported to be less fussy, to get full less easily and rated as enjoying food more than children with lower preferences for these foods. A child may be characterised by their parents as a ‘good eater’ by virtue of their food preferences, which could lead to parents offering a wider variety of fruits and vegetables on more occasions thus reinforcing the child’s preferences further. In all likelihood these associations are complex and bi-directional.

This study has several limitations that require acknowledgement. As discussed, it is not possible to ascertain the direction of the observed associations between appetitive traits and food preferences. All measures were based on parent report and could be subject to bias. It is however worth noting that the CEBQ scales have been shown to have a robust factor structure, good internal reliability [28] and at least three subscales (*enjoyment of food, food responsiveness* and *satiety responsiveness*) have been shown to correlate well with observed behavioural measures of these traits [13]. While food preferences are strong determinants of food intake [48, 49], the diets of small children are ultimately determined by their caregivers. The relationship between children’s dietary intakes and appetite behaviours may differ from the associations between food preferences and appetite behaviours observed here. Future research would benefit from including robust measures of dietary intake as well as food preferences.

Children’s appetitive traits were measured at a separate and earlier time to food preferences in the GEMINI sample. This approximately 2 year time difference may have resulted in an underestimation of the relationships between these variables and contributed to the weaker associations observed between appetitive traits and food preferences in GEMINI compared to NOURISH (where all variables were measured concurrently). GEMINI is a twin cohort and it has been argued that twins are sufficiently different from singletons to preclude generalizations from one to the other [50]. Furthermore, the likelihood of type 1 error occurring with multiple comparisons in a large sample should be acknowledged. The NOURISH sample used in these analyses was relatively small and homogenous; consisting only of first-time mothers, the majority (69 %) of whom held a university level degree. These issues notwithstanding, the replication of results from the large GEMINI twin sample in the smaller NOURISH singleton sample strengthens confidence in the robustness of the study findings.

## Conclusion

This study identified associations between appetitive traits measured at two different ages and preferences for fruits, vegetables and noncore foods in early childhood in two distinct samples. Results do not support convergent positive associations between appetitive traits and food preference patterns thought to be protective against excess weight gain in childhood. Unexpectedly appetitive traits that have been associated with lower obesity risk in childhood were related to lower liking for fruits and vegetables which generally may be expected to increase obesity risk [51, 52]. However *food responsiveness* – a trait associated with higher weight status – was uniquely related to liking for noncore foods. Longitudinal research is needed to uncover causal mechanisms driving associations between appetitive traits and food preferences in early childhood. Taken together, these findings highlight the need to consider dietary quality and variety, as well as overall energy intake and weight, when characterising ‘healthy’ and ‘unhealthy’ appetitive behaviours in children.

## Additional file

Additional file 1: Table S1. Child Eating Behaviour Questionnaire scale items. (DOCX 12 kb)

## References

[1] Olds TS, Tomkinson GR, Ferrar KE, Maher CA (2009). Trends in the prevalence of childhood overweight and obesity in Australia between 1985 and 2008. Int J Obes.

[2] Mandalia D, Craig R, Mindell J (2012). Children’s BMI, overweight and obesity. Health Survey for England 2011: health, social care and lifestyles.

[3] Carnell S, Wardle J (2008). Appetitive traits and child obesity: measurement, origins and implications for intervention. Proc Nutr Soc.

[4] Birch LL (1999). Development of food preferences. Annu Rev Nutr.

[5] Biro FM, Wien M (2010). Childhood obesity and adult morbidities. Am J Clin Nutr.

[6] Cornette R (2008). The emotional impact of obesity on children. Worldv Evid-Based Nu.

[7] Reilly JJ, Methven E, McDowell ZC, Hacking B, Alexander D, Stewart L (2003). Health consequences of obesity. Arch Dis Child.

[8] Must A, Anderson SE (2003). Effects of obesity on morbidity in children and adolescents. Nutr Clin Care.

[9] Lobstein T, Baur L, Uauy R (2004). Obesity in children and young people: a crisis in public health. Obes Rev.

[10] Gallaway MS, Jago R, Baranowski T, Baranowski JC, Diamond PM (2007). Psychosocial and demographic predictors of fruit, juice and vegetable consumption among 11-14-year-old Boy Scouts. Pub Health Nutr.

[11] Russell CG, Worsley A (2007). Do children’s food preferences align with dietary recommendations?. Pub Health Nutr.

[12] Cooke LJ, Wardle J (2005). Age and gender differences in children’s food preferences. Br J Nutr.

[13] Carnell S, Wardle J (2007). Measuring behavioural susceptibility to obesity: validation of the child eating behaviour questionnaire. Appetite.

[14] Bjelland M, Brantsaeter AL, Haugen M, Meltzer HM, Nystad W, Andersen LF (2013). Changes and tracking of fruit, vegetables and sugar-sweetened beverages intake from 18 months to 7 years in the Norwegian Mother and Child Cohort Study. BMC Public Health.

[15] Lien N, Lytle LA, Klepp KI (2001). Stability in consumption of fruit, vegetables, and sugary foods in a cohort from age 14 to age 21. Prev Med.

[16] Northstone K, Emmett PM (2008). Are dietary patterns stable throughout early and mid-childhood? A birth cohort study. Br J Nutr.

[17] Cooke LJ (2007). The importance of exposure for healthy eating in childhood: a review. J Hum Nutr Diet.

[18] Birch LL, Marlin DW (1982). I Dont Like It - I Never Tried It - Effects of Exposure on 2-Year-Old Childrens Food Preferences. Appetite.

[19] Addessi E, Galloway AT, Visalberghi E, Birch LL (2005). Specific social influences on the acceptance of novel foods in 2-5-year-old children. Appetite.

[20] Benton D (2004). Role of parents in the determination of the food preferences of children and the development of obesity. Int J Obesity.

[21] Cashdan E (1994). A Sensitive Period for Learning About Food. Hum Nature-Int Bios.

[22] Cooke LJ, Carnell S, Wardle J (2006). Food neophobia and mealtime food consumption in 4–5 year old children. Int J Behav Nutr Phys Act.

[23] Cooke LJ, Wardle J, Gibson EL (2003). Relationship between parental report of food neophobia and everyday food consumption in 2-6-year-old children. Appetite.

[24] Russell CG, Worsley A (2008). A population-based study of preschoolers’ food neophobila and its associations with food preferences. J Nutr Educ Behav.

[25] Dovey TM, Staples PA, Gibson EL, Halford JC (2008). Food neophobia and ‘picky/fussy’ eating in children: a review. Appetite.

[26] Galloway AT, Fiorito L, Lee Y, Birch LL (2005). Parental pressure, dietary patterns, and weight status among girls who are “picky eaters”. J Am Diet Assoc.

[27] Jacobi C, Agras WS, Bryson S, Hammer LD (2003). Behavioral validation, precursors, and concomitants of picky eating in childhood. J Am Acad Child Psy.

[28] Wardle J, Guthrie CA, Sanderson S, Rapoport L (2001). Development of the Children’s Eating Behaviour Questionnaire. J Child Psychol Psychiatry.

[29] Viana V, Sinde S, Saxton JC (2008). Children’s Eating Behaviour Questionnaire: associations with BMI in Portuguese children. Br J Nutr.

[30] Webber L, Hill C, Saxton J, Van Jaarsveld CH, Wardle J (2009). Eating behaviour and weight in children. Int J Obes.

[31] Spence JC, Carson V, Casey L, Boule N (2011). Examining behavioural susceptibility to obesity among Canadian pre-school children: The role of eating behaviours. Int J Pediatr Obes.

[32] Cooke LJ, Wardle J, Gibson EL, Sapochnik M, Sheiham A, Lawson M (2004). Demographic, familial and trait predictors of fruit and vegetable consumption by pre-school children. Pub Health Nutr.

[33] Rodenburg G, Kremers SPJ, Oenema A, van de Mheen D (2012). Associations of Children’s Appetitive Traits with Weight and Dietary Behaviours in the Context of General Parenting. PLoS One.

[34] Sweetman C, Wardle J, Cooke L (2008). Soft drinks and ‘desire to drink’ in preschoolers. Int J Behav Nutr Phys Act.

[35] van Jaarsveld CH, Johnson L, Llewellyn C, Wardle J (2010). Gemini: a UK twin birth cohort with a focus on early childhood weight trajectories, appetite and the family environment. Twin Res Hum Genet.

[36] Daniels LA, Magarey A, Battistutta D, Nicholson JM, Farrell A, Davidson G (2009). The NOURISH randomised control trial: positive feeding practices and food preferences in early childhood - a primary prevention program for childhood obesity. BMC Public Health.

[37] Wardle J, Sanderson S, Gibson LE, Rapoport L (2001). Factor-analytic structure of food preferences in four-year-old children in the UK. Appetite.

[38] Fildes A, van Jaarsveld CH, Llewellyn CH, Fisher A, Cooke L, Wardle J (2014). Nature and nurture in children’s food preferences. Am J Clin Nutr.

[39] World Health Organization (2006). WHO child growth standards. length/height-for-age, weight-for-age, weight-for-length, weight-for-height and body mass index-for-age. Methods and development.

[40] Llewellyn CH, van Jaarsveld CH, Johnson L, Carnell S, Wardle J (2011). Development and factor structure of the Baby Eating Behaviour Questionnaire in the Gemini birth cohort. Appetite.

[41] Mennella JA, Jagnow CP, Beauchamp GK (2001). Prenatal and postnatal flavor learning by human infants. Pediatrics.

[42] Howard AJ, Mallan KM, Byrne R, Magarey A, Daniels LA (2012). Toddlers’ food preferences. The impact of novel food exposure, maternal preferences and food neophobia. Appetite.

[43] Wardle J, Carnell S, Haworth CM, Farooqi IS, O’Rahilly S, Plomin R (2008). Obesity associated genetic variation in FTO is associated with diminished satiety. J Clin Endocrinol.

[44] Wardle J, Carnell S, Haworth CM, Plomin R (2008). Evidence for a strong genetic influence on childhood adiposity despite the force of the obesogenic environment. Am J Clin Nutr.

[45] Perry RA, Mallan KM, Koo J, Mauch CE, Daniels LA, Magarey AM (2015). Food neophobia and its association with diet quality and weight in children aged 24 months: a cross sectional study. Int J Behav Nutr Phys Act.

[46] Carruth BR, Ziegler PJ, Gordon A, Barr SI (2004). Prevalence of picky eaters among Infants and toddlers and their caregivers’ decisions about offering a new food. J Am Diet Assoc.

[47] Dubois L, Farmer A, Girard M, Peterson K, Tatone-Tokuda F (2007). Problem eating behaviors related to social factors and body weight in preschool children: A longitudinal study. Int J Behav Nutr Phys Act.

[48] Drewnowski A, Hann C (1999). Food preferences and reported frequencies of food consumption as predictors of current diet in young women. Am J Clin Nutr.

[49] Birch L (1979). Preschool children’s food preferences and consumption patterns. J Nutr Educ.

[50] Evans DM, Martin NG (2000). The validity of twin studies. GeneScreen.

[51] Howarth NC, Saltzman E, Roberts SB (2001). Dietary fiber and weight regulation. Nutr Rev.

[52] Rolls BJ, Ello-Martin JA, Tohill BC (2004). What can intervention studies tell us about the relationship between fruit and vegetable consumption and weight management?. Nutr Rev.

