# Supplementary material for: The relationship between appetite and food preferences in British and Australian children
Source: Int J Behav Nutr Phys Act. 2015 Sep 17;12:116. doi: 10.1186/s12966-015-0275-4 (PMC4574466; doi:10.1186/s12966-015-0275-4)
Supplement: Additional file 1: Table S1. — Child Eating Behaviour Questionnaire scale items. (DOCX 12 kb) [file 12966_2015_275_MOESM1_ESM.docx]

Supplementary table: Child Eating Behaviour Questionnaire scale items:

| **Enjoyment of Food^a^** | **Food Responsiveness ^a^** | **Satiety Responsiveness ^a^** | **Slowness in Eating ^a^** | **Food Fussiness ^a^** |
| --- | --- | --- | --- | --- |
| My child loves food | My child is always asking for food | My child has a big appetite^b^ | My child finishes his/her meal quickly^b^ | My child refuses new foods at first |
| My child is interested in food | If allowed to, my child would eat too much | My child leaves food on his/her plate at the end of a meal | My child eats slowly | My child enjoys tasting new foods^b^ |
| My child looks forward to mealtimes | Given the choice, my child would eat most of the time | My child gets full before his/her meal is finished | My child takes more than 30 minutes to finish a meal | My child enjoys a wide variety of foods^b^ |
| My child enjoys eating | Even if my child is full up s/he finds room to eat his/her favourite food | My child gets full up easily | My child eats more and more slowly during the course of a meal | My child is difficult to please with meals |
|  | If given the chance, my child would always have food in his/her mouth^c^ | My child cannot eat a meal if s/he has had a snack just before |  | My child is interested in tasting food s/he hasn’t tasted before^b^ |
|  |  |  |  | My child decides that s/he doesn’t like a food, even without tasting it |

**^a^** Items were scored on a 5-point scale as ‘never’, ‘rarely’, ‘sometimes’, ‘often’, or ‘always’. Mean scores were calculated for each subscale (range: 1–5) with higher scores indicating higher values of each trait. In order to calculate scale scores complete data was required on a minimum of 60% of scale items.

**^b^** Scores were reversed for these items

**^c^** This item was not included in the adapted toddler version of the CEBQ completed by the GEMINI sample. All other items and scales used in this study were unchanged.
